# Short-Term Environmental Enrichment Rescues Adult Neurogenesis and Memory Deficits in APP_Sw,Ind_ Transgenic Mice

**DOI:** 10.1371/journal.pone.0016832

**Published:** 2011-02-09

**Authors:** Jorge Valero, Judit España, Arnaldo Parra-Damas, Elsa Martín, José Rodríguez-Álvarez, Carlos A. Saura

**Affiliations:** Institut de Neurociències, Department de Bioquímica i Biologia Molecular, Centro de Investigación Biomédica en Red Enfermedades Neurodegenerativas (CIBERNED), Universitat Autònoma de Barcelona, Barcelona, Spain; Sapienza University of Rome, Italy

## Abstract

Epidemiological studies indicate that intellectual activity prevents or delays the onset of Alzheimer's disease (AD). Similarly, cognitive stimulation using environmental enrichment (EE), which increases adult neurogenesis and functional integration of newborn neurons into neural circuits of the hippocampus, protects against memory decline in transgenic mouse models of AD, but the mechanisms involved are poorly understood. To study the therapeutic benefits of cognitive stimulation in AD we examined the effects of EE in hippocampal neurogenesis and memory in a transgenic mouse model of AD expressing the human mutant β-amyloid (Aβ) precursor protein (APP_Sw,Ind_). By using molecular markers of new generated neurons (bromodeoxiuridine, NeuN and doublecortin), we found reduced neurogenesis and decreased dendritic length and projections of doublecortin-expressing cells of the dentate gyrus in young APP_Sw,Ind_ transgenic mice. Moreover, we detected a lower number of mature neurons (NeuN positive) in the granular cell layer and a reduced volume of the dentate gyrus that could be due to a sustained decrease in the incorporation of new generated neurons. We found that short-term EE for 7 weeks efficiently ameliorates early hippocampal-dependent spatial learning and memory deficits in APP_Sw,Ind_ transgenic mice. The cognitive benefits of enrichment in APP_Sw,Ind_ transgenic mice were associated with increased number, dendritic length and projections to the CA3 region of the most mature adult newborn neurons. By contrast, Aβ levels and the total number of neurons in the dentate gyrus were unchanged by EE in APP_Sw,Ind_ mice. These results suggest that promoting the survival and maturation of adult generated newborn neurons in the hippocampus may contribute to cognitive benefits in AD mouse models.

## Introduction

Adult neurogenesis is a continuous physiological process that occurs in the dentate gyrus (DG) of the hippocampus and the subventricular zone in rodents and primates, including humans [1]–[4]. The majority of newborn neurons die within the first four weeks after birth, whereas few of them survive and integrate into deeper layers of the DG granular cell layer (GCL) [5]–[7]. During the critical maturation stage (1–4 weeks), newborn neurons undergo dendritic morphological changes, project their axons to the CA3 region and integrate functionally into hippocampal neural circuits [8]–[11]. There is strong evidence indicating that adult neurogenesis contributes to hippocampal-dependent learning and memory [9], [12]–[15]. For instance, factors that increase adult neurogenesis, such as environmental enrichment (EE) and exercise, improve memory function (for review, see [16], [17]), whereas a reduction of neurogenesis induced by stress results in memory impairment [18], [19]. Notably, activity-induced stimulation and morphological changes of newborn immature neurons during the maturation stage are critical for their functional integration into hippocampal memory circuits [9], [12], [15], [20].

Altered adult neurogenesis has been suggested to contribute to cognitive decline in normal and pathological aging including Alzheimer's disease (AD) [21]–[23]. AD, the most common neurodegenerative disorder, is characterized by cognitive decline and neuropathological lesions that include β-amyloid (Aβ) plaques, neurofibrillary tangles and neuronal loss in the brain. Accumulation of neurotoxic Aβ peptides in the hippocampus and cerebral cortex is thought to contribute to synaptic dysfunction in AD [24]. Studies of adult neurogenesis in AD have provided contradictory results revealing either decreased or increased adult neurogenesis in transgenic mice overexpressing the β-amyloid precursor protein (APP). Thus, unchanged or increased production of neural progenitors after the proliferative stage (1 week) and before the survival period has been detected in different APP transgenic mice [25]–[29]. However, reduced number of adult newborn neurons as a result of diminished survival was recently reported in APP transgenic mice [27], [29]–[32]. Similarly, cognitive stimulation and physical activity have differential effects in neurogenesis and memory in AD mice, which are likely due to differences in animal models and EE conditions. Long-term EE for at least 4 months ameliorates learning and memory deficits in AD transgenic mouse models [29], [33]–[36]. The behavioural benefits of long-term cognitive stimulation in APP mice has been associated with increased or unchanged number of cells expressing calretinin, a late marker of immature neuronal precursors, rather than changes on proliferation of adult newborn neurons [34], [37]. By contrast, EE has no benefits on cognition and neurogenesis in transgenic mice with an AD-like aggressive phenotype [38]. In these previous studies mice were exposed to EE conditions for at least four months and during manifestations of AD-like pathology.

Here, we examined the effect of short-term enrichment on memory performance and its relationship to survival and maturation of adult newborn neurons in wild-type (WT) and APP_Sw,Ind_ mice at early pathological stages. We found that short-term EE for 7 weeks efficiently improves early hippocampal-dependent spatial learning and long-term memory in APP_Sw,Ind_ transgenic mice. The cognitive benefits of EE were associated with increased neurogenesis and dendritic morphology and projections of adult newborn neurons in the DG. These results suggest that promoting the survival and maturation of adult generated newborn neurons in the hippocampus may contribute to cognitive benefits in AD mouse models.

## Materials and Methods

### Mice and environmental enrichment protocol

APPSw,Ind transgenic mice (line J9; C57BL/6 background) expressing the mutant human APP695 isoform harboring the FAD-linked Swedish (K670N/M671L)/Indiana (V717F) mutations under the expression of the neuronal PDGFβ promoter were previously described [39], [40]. For all experiments, we used age-matched littermate female mice obtained from heterozygous APP_Sw,Ind_ x non-transgenic (WT) crossings. Thirty-one female non-transgenic WT (control) and twenty-seven female APP_Sw,Ind_ transgenic mice were used for this study. All mice were housed in the same room and kept on a 12 h light/dark schedule and given *ad libitu*m access to food and water.

Mice at 4 months of age were placed into large enrichment cages (EE, 6–8 animals per cage with a floor surface of 1875 cm^2^) or standard housed in normal size cages (6 animals per cage with a floor surface of 500 cm^2^). The enriched cages contained plastic play tubes, 2–4 shelters, and one running wheel (Supplemental Fig. S1) that were changed or rearranged every five days to provide novel stimulation [16], [17].

### Bromodeoxyuridine administration

To label proliferating cells, the thymidine analog 5-bromo-2′-deoxyuridine (BrdU; Sigma Chemical Co., St. Louis, MO) was administered (200 mg/Kg twice per day) intraperitoneally in 0.1 M phosphate buffered-saline (PBS) at pH 7.3 for 5 days, [41]. Animals were injected with BrdU one week after placing them in EE conditions and sacrificed ∼7 weeks after the last BrdU injection.

### Morris water maze test

The Morris water maze (MWM) consisted of a circular pool (120 cm in diameter) containing a hidden platform (11 cm in diameter) submerged 0.5 cm below opaque water (21±2°C). The pool was enclosed with black curtains and surrounded by four different visual cues. For each trial, mice were placed into the pool at one of four starting points in a pseudorandom order. Mice were trained for 5 days (six trials per day) with maximum trial duration of 60 s and an intertrial interval of 15 min. To assess memory retention mice were tested in a 1 min-probe trial without the platform 2.5 h after training on day 5 [42]. Performance on the water maze task was analyzed by the *SMART* software (PanLab S.L., Barcelona, Spain). The experimenter was blind to the genotypes and groups of mice.

### Immunofluorescence staining

Mice were deeply anesthetized with 40 µl/g b.w. of a mixture of ketamine hydrochloride (0.9 ml; Imalgene 1000, Merial, France) and Xilacine (0.5 ml, Rompun 2%, Bayer, Germany), and then perfused intracardially with 0.9% NaCl followed by 4% (w/v) buffered paraformaldehyde. Cerebral hemispheres were postfixed with the same fixative for 2 h, rinsed with PBS for 2 h and immersed in 30% (w/v) sucrose. After cryoprotection, 40 µm-thick coronal sections were obtained with a criostat (Leica, Mannheim, Germany).

For triple BrdU/NeuN/c-fos staining, sections were rinsed in PBS, incubated in 1 N HCl (1 h) to allow DNA denaturation and rinsed in 0.1 M borate (pH 8.5) and PBS buffer. Sections were incubated with rat anti-BrdU (1∶2,000; ab6326, Abcam, New Zeland), mouse anti-NeuN (1∶4,000; MAB377, Chemicon, USA), rabbit anti-c-fos (1∶1,000; ab7963-1, Abcam) in PBS containing 0.2% Triton X-100 (v/v) and 5% normal goat serum for 16 h at 4°C. For doublecortin (Dcx) immunofluorescence detection we used the same protocol but omitting HCl and borate buffer incubations. Tissue was incubated with rabbit anti-Dcx (1∶1,000; ab18723, Abcam) for 48 h. Sections were incubated with the corresponding secondary antisera: Alexa488-conjugated goat anti-rat IgG, Alexa594-conjugated goat anti-rabbit IgG or Alexa680-conjugated goat anti-mouse IgG (all of them at 1∶400 from Invitrogen). Sections were counterstained with Hoechst (1∶10,000; Invitrogen) and mounted with FluoroSave Reagent (Calbiochem).

### Cell number and volume quantifications

Quantification of BrdU positive cells along the entire granule cell layer (GCL) of the DG was performed as previously described [41]. The areas of the GCL were measured in serial sections at 240 µm rostrocaudal intervals (11 to 15 sections per animal) along the entire hippocampus using the ImageJ 1.42q software (Wayne Rasband, National Institutes of Health, USA), whereas the volume of the GCL was estimated with the TableCurve 2D v5.01 software (AISN Software, Mapleton, USA). The density of BrdU cells in the GCL was obtained by counting the number of BrdU cells in the GCL of one in every six sections (n = 6–8 mice/group). The total number of BrdU, Dcx and NeuN cells in the DG were calculated by using the Abercrombie-based method as previously reported [43], [44]. Density of Dcx cells in the entire GCL was quantified in six sections per animal at similar rostrocaudal levels (from bregma −2.00 mm to −3.00) using an epifluorescent microscope (Nikon Eclipse 90i). Estimation of total number of double stained BrdU/NeuN cells was performed by calculating the percentage of BrdU positive cells expressing NeuN. This analysis was performed using confocal images from coronal sections at similar rostro-caudal levels (from bregma −1.70 mm to −3.00 mm) obtained with a Leica TCS SP5 laser confocal microscopy by using the Leica Application Suite (Advanced Fluorescente Lite 1.8.1).

### Dendritic morphology analysis

For analyses of dendritic morphology, we obtained 3D reconstructions of the dendritic tree of the most mature Dcx cells (n≥8 cells/mouse, n = 6 mice/group) by using the Simple neurite tracer plugin (http://homepages.inf.ed.ac.uk/s9808248/imagej/tracer/) and the image processing package Fiji (http://pacific.mpi-cbg.de/wiki/index.php/Main_Page) in images captured with confocal microscopy. Selection of cells was based on dendritic morphological description of Dcx-positive EF type cells and previously established criteria [45], [46]. Dendritic morphology was analyzed using the Sholl analysis. The log-log Sholl analysis was used to obtain the Sholl's regression coefficient [47], [48]. We also, compared the number of intersections at each given radius (steps of 10 µm) and number of branching and endings into each given circle (radii of circles increasing at regular steps of 50 µm). For analysis of Dcx fibers, images of three equivalent sections per mouse (from bregma −1.70 to −2.20; n = 6 mice/group) were processed using ImageJ software as previously described [49]. Percentage of Dcx-stained area in three CA3 regions (proximal, medial and distal relative to the DG) was determined using three different squared areas (70×70 µm) in the stratrum lucidum.

### Aβ measurement

Mouse hippocampi were dissected out and snap-frozen. Total levels of Aβ40 and Aβ42 in mouse hippocampus (n = 4 mice/group) were determined by using the hAmyloid β40 and β42 brain ELISA kits following the manufacturer instructions (The Genetics company, Schlieren, Switzerland).

### Statistical analysis

Statistical analyses were performed with the two-tailed unpaired Student *t* test for differences between two means. For multiple comparisons, we used one- or two-way analysis of variance (ANOVA) followed by the Student-Newman-Keuls post hoc test. Correlations were examined by linear regression analysis. Differences with *p*<0.05 were considered significant.

### Ethics Statement

Animal experimental procedures were performed in accordance with institutional and national guidelines following approval by the Animal Care and Ethical Committee of the Universitat Autònoma de Barcelona (protocol CEEA 475, DMAH 3936).

## Results

### Short-term environmental enrichment improves learning and long-term memory independently of Aβ changes in APP_Sw,Ind_ mice

**Figure 1:**
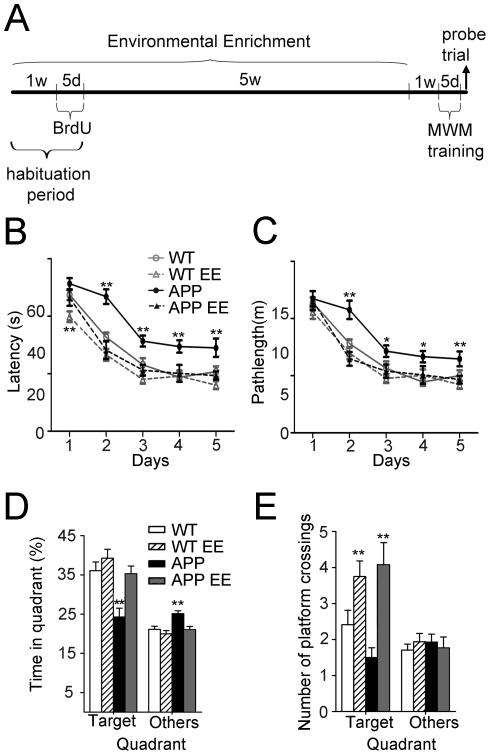
Short-term environmental enrichment improves learning and memory in young APP_Sw,Ind_ mice. (A) The experimental design consisted of 7 weeks of environmental enrichment (EE), that included 5 days BrdU injections, and 1 week without enrichment followed by the Morris water maze (MWM). (B,C) The performance of WT (n = 17), APP_Sw,Ind_ (n = 14) and 7 week-enriched WT (n = 16) and APP_Sw,Ind_ (n = 14) mice at the age of 6 months improved significantly during the training days in the hidden version of the MWM, indicating that all groups were able to learn the task. Compared to WTs, APP_Sw,Ind_ mice, but not enriched APP_Sw,Ind_ mice, exhibited significantly longer latencies and pathlength from training day 2 to 5. (D) Percentage of time spent in the target and other quadrants during the probe trial on day 5 in the MWM. WT and APP_Sw,Ind_ EE mice displayed significantly higher occupancy of the target quadrant relative to other quadrants (*p*<0.0001). (E) Number of platform crossings during the probe trial. EE increases the number of target platform crossings in both WT and APP_Sw,Ind_ mice compared to standard housed mice. Significant differences were analyzed by two-way ANOVA. Data represent mean ± SEM. **p<0.05, **p<0.01, compared to WT mice.

APP_Sw,Ind_ transgenic mice show hippocampal-dependent learning and memory deficits associated with accumulation of intraneuronal Aβ in the DG, CA1 and CA3 hippocampal regions at 6 months of age [40], [42]. To investigate whether short-term environmental enrichment (EE) had therapeutic benefits in AD, APP_Sw,Ind_ mice at 4 months of age were housed in standard or EE conditions for 7 weeks and then tested for memory performance in the MWM (Fig. 1A and Supplemental Fig. S1). The performance of WT and APP_Sw,Ind_ mice improved significantly during the training days (day 1 versus day 5, *p<*0.001) (Fig. 1B). Statistical analyses revealed a significant main effect of genotype/housing (Two-way ANOVA, genotype/housing effect: F_(3,224)_  = 33.58; day effect: F_(4,224)_  = 104.59; *p<*0.0001). Compared to WT mice, standard housed APP_Sw,Ind_ mice, but not APP_Sw,Ind_ EE mice, exhibited significantly longer latencies and pathlengths from training day 2 to 5 (Fig. 1B–C). In the probe trial, standard housed WT and enriched WT or APP_Sw,Ind_ groups displayed significantly higher permanencies in the target quadrant compared to other platform locations (*p<*0.0001), whereas standard housed APP_Sw,Ind_ mice failed to show such a preference (*p>*0.05) (Fig. 1D). Similarly, EE increased significantly the number of target platform crossings in WT and APP_Sw,Ind_ mice compared to standard housed mice (*p*<0.01; Fig. 1E). These results demonstrate that short-term EE prevents hippocampal-dependent spatial learning and memory impairments in young APP_Sw,Ind_ mice.

Since long-term EE for 3–6 months affects Aβ levels in APP transgenic mice [29], [33], [36], [50], we next quantified the levels of Aβ40 and Aβ42 peptides in the hippocampus of the experimental groups. Notably, total levels of Aβ40 and Aβ42 were similar in the hippocampus of standard caged and EE APP_Sw,Ind_ mice (APP_Sw,Ind_, Aβ40: 1.83±0.34 pmol/g, Aβ42: 0.81±0.16 pmol/g; APP_Sw,Ind_ EE, Aβ40: 2.15±0.28 pmol/g, Aβ42: 0.77±0.17 pmol/g; *p*>0.05). Taken together, these results indicate that short-term EE reverses early hippocampal-dependent spatial learning and memory impairments independently of Aβ changes in APP_Sw,Ind_ mice.

### Environmental enrichment increases adult neurogenesis in APP_Sw,Ind_ mice

**Figure 2:**
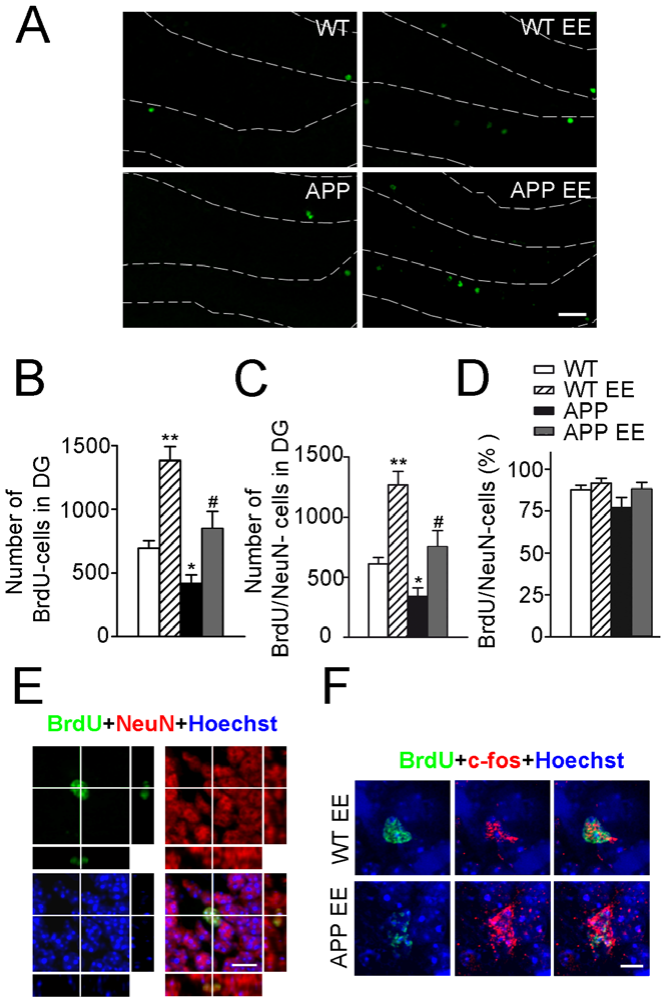
Environmental enrichment increases adult neurogenesis in APP_Sw,Ind_ mice. (A) Confocal microscopy images showing BrdU (green) positive cells in the granule cell layer (GCL; dashed lines). Scale bar  = 40 µm. (B) Neurogenesis was assessed by counting the number of BrdU-stained cells in one of six sections throughout the whole rostro-caudal axis of the DG and estimating the total number of BrdU cells according to the DG volume. Total number of BrdU cells is significantly decreased in the DG of APP_Sw,Ind_ mice whereas environmental enrichment (EE) increases significantly the number of BrdU cells in APP_Sw,Ind_ mice. (C) Total number of newly-generated neurons (BrdU/NeuN cells) was significantly reduced in APP_Sw,Ind_ mice and increased by EE. (D) No significant differences in the percentage of BrdU/NeuN-cells are detected among the experimental groups indicating that EE recovers normal levels of adult neurogenesis without altering the rate of neuronal differentiation in APP_Sw,Ind_ mice. (E) Confocal microscopy images of the GCL of a WT mouse stained for BrdU (green), NeuN (red), and counterstained with Hoechst (blue). Lines intersect in a double stained cell and indicate orthogonal planes showed in the right (y-z axis) and bottom (x-z axis). Scale bar  = 10 µm. (F) Confocal microscopy images of the GCL of the dentate gyrus showing a single (WT EE) or two (APP EE) neurons triple stained for BrdU (green), c-fos (red) and Hoeschst (blue). Scale bar  = 5 µm. Data represent mean ± SEM. *p<0.05 and **p<0.01, compared to WT mice. ^#^ *p*<0.05 compared to APP_Sw,Ind_ mice. Number of mice: WT (n = 8), WT EE (n = 7), APP_Sw,Ind_ (n = 6) and APP_Sw,Ind_ EE (n = 6).

To study whether improvement of spatial memory as a result of short-term EE was related to changes on adult neurogenesis in APP_Sw,Ind_ mice we labelled newly-generated cells using the extrinsic cell proliferation marker BrdU. BrdU was administered during the enriched habituation period (Fig. 1A). To identify newly generated neurons incorporated to the DG circuitry [5]–[7] we used double immunofluorescence staining for BrdU and NeuN, a marker of mature neurons, or c-fos, a marker of neuronal activity [9]. Quantitative analysis revealed that total number of BrdU and double BrdU/NeuN-stained neurons were significantly decreased (∼40%) in the DG of APP_Sw,Ind_ mice (Student-Newman-Keuls post hoc test: *p<*0.05; Fig. 2A–D). Interestingly, EE increased significantly the number of BrdU and BrdU/NeuN cells (∼2-fold) both in WT and APP_Sw,Ind_ mice compared with standard housed mice (*p<*0.05; Fig. 2A,D). Two-way ANOVA revealed no statistical differences in the percentage of BrdU-cells stained for NeuN among the different experimental groups (genotype/housing interaction effect: F_(1,23)_  = 0.89, *p* = 0.35, genotype effect: F_(1,23)_  = 3.30, *p* = 0.0822; housing effect: F_(1,23)_ = 3.65, *p* = 0.07). Importantly, we detected cells double stained for BrdU and c-fos in EE WT and APP mice (Fig. 2F), indicating that newborn neurons were efficiently recruited into spatial memory circuits. These results indicate that short-term EE enhances adult neurogenesis without affecting the rate of neuronal differentiation in APP_Sw,Ind_ mice.

### Environmental enrichment increases the number and dendritic length of mature doublecortin cells in APP_Sw,Ind_ mice

**Figure 3:**
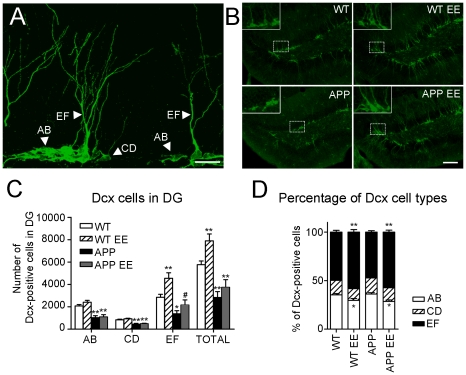
Environmental enrichment enhances the population of most-mature doublecortin cells in APP_Sw,Ind_ mice. (A) Confocal microscopy image (maximum projection of several planes) of the dentate gyrus (DG) of an enriched WT mouse stained with doublecortin (Dcx) antibody showing the morphology of proliferative (type AB), intermediate (type CD) and postmitotic (type EF) neurons. Scale bar  = 20 µm. (B) Representative epifluorescence microscopy images showing Dcx positive cells in the DG of the four experimental groups. Magnified images are shown in the insets. Scale bar  = 100 µm. (C) Quantitative analysis reveals a significant reduction of total number of Dcx-labelled cells in the DG of APP_Sw,Ind_ mice (n = 6 mice) compared with WT mice (n = 6 mice). The number of EF-type neurons, but not AB- and CD-type neurons, is significantly increased in both WT (n = 6 mice) and APP_Sw,Ind_ mice (n = 6 mice) after EE. (D) Both enriched WT and APP_Sw,Ind_ mice show a significant increase in the percentage of EF cells and reduced percentage of AB cells compared to standard housed mice. Data represent mean ± SEM. *p<0.05 and **p<0.01, compared to WT mice. # *p*<0.05, compared to APP mice.

**Figure 4:**
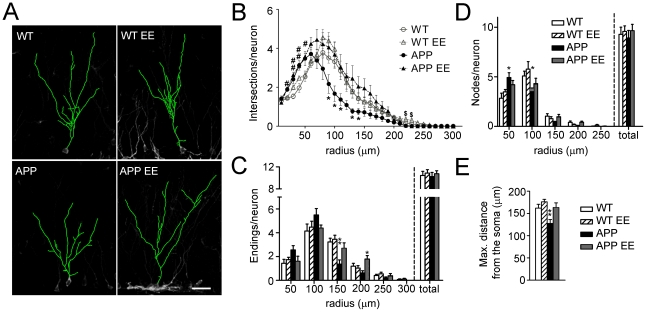
Normal morphology of doublecortin-positive neurons after environmental enrichment in APP_Sw,Ind_ mice. (A) Three dimensional reconstructions of doublecortin (Dcx) stained cells (green) in the DG region of the hippocampus of the different experimental groups. Scale bar  = 30 µm. (B) At least eight EF type Dcx-cells per mouse (n = 6 mice/group) were reconstructed using confocal images and the number of intersections of dendrites with concentrical circles at increasing radius (10 µm) were quantified. Reduced number of intersections from radius 90–140 in APP_Sw,Ind_ mice was efficiently reverted by environmental enrichment (EE). (C) Number of dendritic endings into the area delimited by concentric circles at increasing radius (50 µm). Decreased number of neuronal dendritic endings at 150 µm from the soma in APP_Sw,Ind_ mice is reversed by EE. (D) The number of nodes, which is significantly increased at 50 µm and reduced at 100 µm from the soma in APP_Sw,Ind_ mice, is normalized by enrichment. (E). The maximum distance reached by the dendritic tree of Dcx+ cells into the molecular layer of the DG was significantly reduced in APP_Sw,Ind_ mice and reversed by EE. Data represent mean ± SEM. In B: ^#^ *p*<0.05 and ^##^ *p*<0.01, compared to WT and WT EE mice; *p<0.05, compared to the rest of groups; ^$^ *p*<0.05, WT EE mice versus the other groups. In C, D and E: *p<0.05 and **p<0.01, APP_Sw,Ind_ mice or APP_Sw,Ind_ EE mice versus the other groups. Number of cells/mouse: ≥8; Number of mice/group: 6.

EE has been reported to increase proliferation, survival and maturation of newborn neurons [51], [52]. We then analyzed the number and morphology of neurons expressing Dcx, a cytoskeletal factor expressed in neuroblasts from early neuronal differentiation stages to 28 days after birth. Dcx staining allowed us to analyze newborn neurons in the critical maturation period, which has been recently proposed to contribute to spatial learning and memory [12], [15]. Dcx cells have been previously categorized in six different types (named A–F) depending on the dendritic morphology and proliferative (EF), intermediate (CD) or postmitotic (EF) stages (Fig. 3A) [46]. In agreement with the BrdU counting (Fig. 2B), the total number of Dcx labelled cells in the DG of APP_Sw,Ind_ mice was significantly reduced (∼50%) compared to WT mice (Student-Newman-Keuls post hoc test: *p<*0.01; Fig. 3B and C). EE increased the number of Dcx cells in WT mice (40%, *p<*0.01; Fig. 4), whereas it had minor effects on APP_Sw,Ind_ mice. Although EE did not change the total number of AB- and CD-type cells, it increased significantly the number of EF-type cells in both WT and APP_Sw,Ind_ mice (Fig. 3C; *p*<0.05). Enriched WT and APP_Sw,Ind_ mice showed a significant reduction in the percentage of AB-type cells, whereas the percentage and number of EF-type cells were significantly increased compared to standard housed mice (*p*<0.05; Fig. 3D). In agreement with the BrdU/NeuN staining results (Fig. 2C), the number of EF-type cells in the DG of enriched APP_Sw,Ind_ mice was not significantly different from standard-housed WT mice and increased compared to standard-housed APP_Sw,Ind_ mice (*p>*0.05; Fig, 3C). These results indicate that environmental stimulation increases the population of most mature Dcx-positive neurons in WT and APP_Sw,Ind_ mice.

Dendritic morphology of DG granule cells is altered in transgenic mouse models of AD [53], [54]. We then examined whether cognitive stimulation with EE would result on dendritic morphology changes in EF-type Dcx-positive cells. To address whether dendritic and branching morphology was altered in APP_Sw,Ind_ mice we first relied on the commonly used Sholl analysis. This method consisted of drawing consecutive circles around the soma of the cells and quantifying dendritic arborisation pattern by cumulative intersections [47], [48]. Determination of the Sholl regression coefficient in EF-type cells, which is considered as a general index of neuronal morphology [47], showed no significant differences between the experimental groups indicating that the EF-type Dcx cells showed similar dendritic morphologies (WT: 2.05±0.02, WT EE: 2.02±0.04, APP_Sw,Ind_: 2.00±0.04, APP_Sw,Ind_ EE: 2.00±0.05; F_(3,20)_  = 0.49; *p>*0.05). Analysis of circle intersections at different distances (0–250 µm) revealed a significant increase in the number of short-distance dendrites (radius 20–50 µm) in standard housed and enriched APP_Sw,Ind_ mice and a decrease in the number of long-distance dendrites (radius 90–140 µm) in standard housed APP_Sw,Ind_ mice. Notably, enrichment enhanced the number of dendrites at long distances in APP_Sw,Ind_ mice (Fig. 4A–B). In agreement with these data, the reduced number of dendritic endings of Dcx neurons at long distances (150–200 µm) was reversed by EE in APP_Sw,Ind_ mice (Fig. 4C). Changes in the branching pattern indicated by the increase in the number of nodes at 50 µm and their reduction at 100 µm from the soma were also reversed by enrichment in APP_Sw,Ind_ mice (Fig. 4D). Importantly, the total number of endings and nodes of Dcx cells was similar in all experimental groups (Fig. 4C–D), indicating that reduced dendritic complexity of EF-type Dcx cells in APP_Sw,Ind_ mice was due to the fact that dendrites end and branch closer from the soma. In fact, EE reverses the decrease in the maximum distance of dendrites from the soma of Dcx-positive cells in APP_Sw,Ind_ mice (Student-Newman-Keuls post hoc test: *p<*0.01, Fig. 4E). Taken together, these data indicate that EE reverses efficiently the deficits in the number and dendritic morphology of mature newborn neurons in the DG of APP_Sw,Ind_.

### Environmental enrichment enhances density of doublecortin positive fibers in the CA3 region of APP_Sw,Ind_ mice

**Figure 5:**
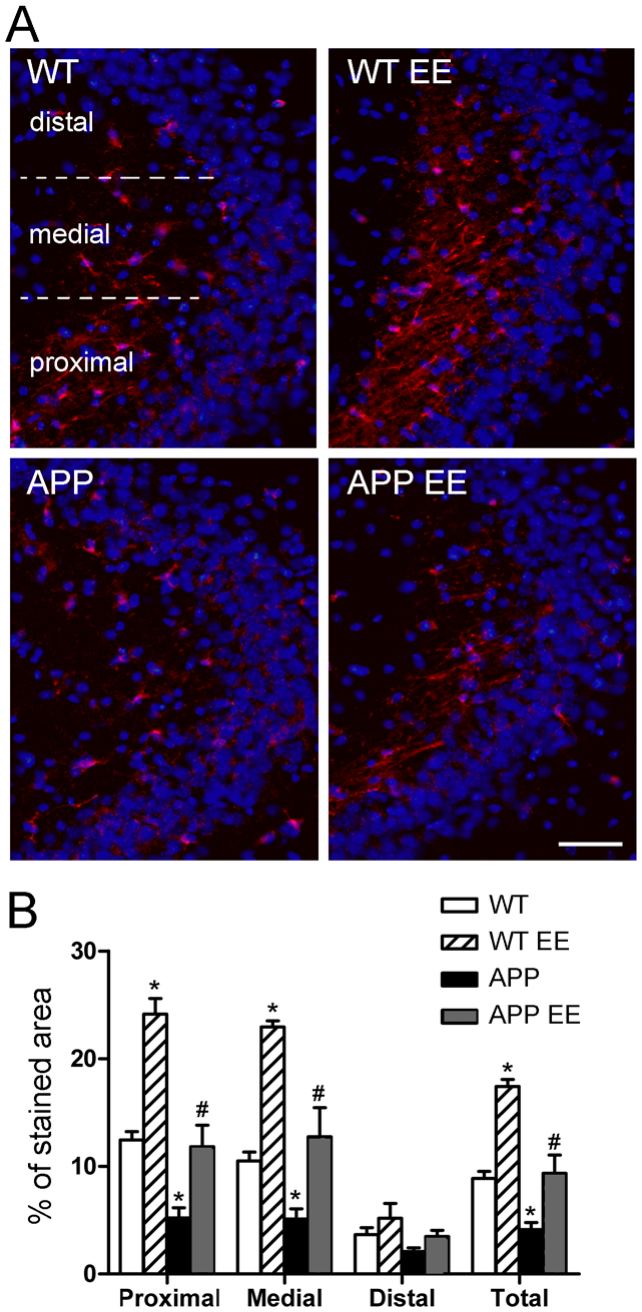
Density of doublecortin-positive fibers in CA3 hippocampal region of control and APP_Sw,Ind_ mice. (A) Representative epifluorescence microscopy images of the stratum lucidum stained for doublecortin (Dcx, red) and Hoechst (blue). Scale bar  = 50 µm. (B). Semiquantitative analysis reveals a significant reduction in the area ocuppied by Dcx-positive fibers in the stratum lucidum of the CA3 region in APP_Sw,Ind_ mice. Enrichment increased significantly the density of Dcx staining in the stratum lucidum in both WT and APP_Sw,Ind_ mice compared to standard housed mice. Data represent mean ± SEM. *p<0.01, compared to WT mice. ^#^ *p*<0.01, compared to APP_Sw,Ind_ mice. Number of mice/group: 6.

Axonal projections of adult newborn neurons can reach the stratum lucidum of the CA3 hippocampal region as early as 4–10 days after their birth [8], [55]. Since Dcx staining in the stratum lucidum of the hippocampus reflects the projection of DG newly-generated neurons into the CA3 layer, we quantified the area occupied by Dcx fibers in proximal, medial and distal regions of the stratum lucidum related to DG. In agreement with a decrease in the number and dendritic morphology of the most mature EF-type Dcx cells, we found a significant reduction (∼54%) of Dcx-positive staining in the CA3 stratum lucidum in APP_Sw,Ind_ mice (Student-Newman-Keuls post hoc test: *p*<0.01; Fig. 5). Compared with standard housed mice, EE significantly increased the projections of Dcx-positive fibers specifically in proximal and medial regions of CA3 in WT and APP_Sw,Ind_ mice (*p<*0.01; Fig. 5). These results suggest that EE enhances the mossy fiber projections of adult newborn neurons in APP_Sw,Ind_ mice.

### APP_Sw,Ind_ mice show reduced number of neurons in the dentate gyrus

**Figure 6:**
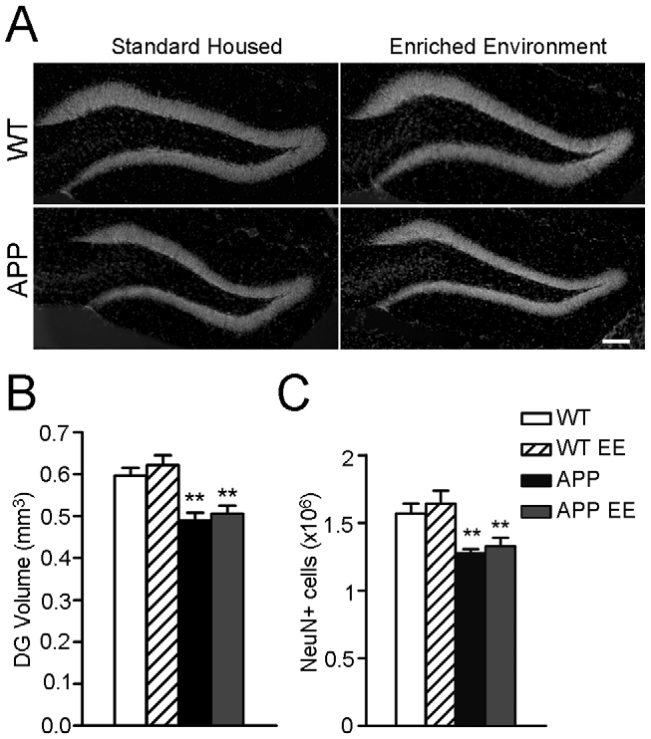
APP_Sw,Ind_ mice show reduced neurons in the dentate gyrus. (A) Representative epiflurescence microscopy images of the granule cell layer (GCL) of the dentate gyrus counterstained with Hoechst. Reduced thickness of the GCL is evident in standard housed and enriched APP_Sw,Ind_ mice. Scale bar  = 100 µm. (B) Quantitative analysis show a significant reduction of GCL volume in APP_Sw,Ind_ mice. (C) Total number of neurons stained for NeuN in the GCL of standard housed and enriched (EE) APP_Sw,Ind_ mice was significantly reduced compared with WT mice. Data represent mean ± SEM. *p<0.05; **p<0.01, compared to WT mice. Number of mice: WT (n = 8), enriched WT (n = 7), APP_Sw,Ind_ (n = 6) and enriched APP_Sw,Ind_ (n = 6).

The incorporation of newly generated neurons in the DG has been postulated to increase the cell number and volume of the DG during the first 8 months of age [56], [57]. I*n vivo* and postmortem analysis of brains of AD patients have revealed significant neuronal and volume loss in the DG [58]. To examine whether sustained decrease of adult neurogenesis could lead to reduced number of total mature neurons and/or volume of the GCL of the hippocampus in APP_Sw,Ind_ mice, we next estimated the number of NeuN-positive (mature) neurons and volume of the DG. Interestingly, imaging and quantitative analyses revealed a low but significant reduction (∼17%) in the number of NeuN cells and volume of the DG (∼17%) in APP_Sw,Ind_ mice (Student-Newman-Keuls post hoc test: *p*<0.01). However, enrichment was not able to reverse these morphological changes in APP_Sw,Ind_ mice (Fig. 6). These results suggest that extended reduction of adult neurogenesis may lead to decreased number of granule cells and volume of the DG in APP_Sw,Ind_ mice.

## Discussion

Epidemiological studies indicate that cognitive stimulation resulting from intellectual and physical activities prevents or delays the onset of AD [59]–[64], whereas cognitive training improves memory function in AD patients at early-disease stages [65]–[67]. Similarly, long-term EE improves learning and memory in AD transgenic mice during the development of amyloid plaque pathology, but the role of neurogenesis in this process has not been clearly discerned [33]–[37]. In this study, we evaluated the effects of short-term EE in APP_Sw,Ind_ mice at 4 months, an age characterized by subtle spatial memory deficits and increased hippocampal levels of Aβ but prior to amyloid deposition [39], [42], [68]. This experimental design allowed us to examine the therapeutic benefits of acute environmental stimulation in memory and neurogenesis during the course of early pathological stages.

Several studies have shown impaired neurogenesis in APP transgenic mice [27], [29]–[32], [69], [70], which has lead to the hypothesis that an enhancement of neurogenesis might have therapeutic benefits in AD [21], [58]. Our results revealed that short-term EE efficiently rescues hippocampal-dependent learning and memory deficits in young APP_Sw,Ind_ mice. The improvement of memory function was associated with increased adult neurogenesis, normal dendritic morphology and CA3 innervation of adult generated neurons in the DG. Adult neurogenesis has been positively associated with spatial learning and memory performance [9], [12]–[14], [20], [22], [71], [72], although adult neurogenesis is not essential to maintain spatial memory in certain circumstances [73]–[75]. We detected newly generated neurons expressing c-fos, a marker of neuronal activity, after the spatial memory test indicating a functional integration of newborn neurons into spatial memory circuits [9]. Moreover, spatial memory index correlated tightly with the number of BrdU/NeuN cells in APP_Sw,Ind_ mice (Supplemental Fig. S2), whereas such a correlation was not found in non-transgenic mice. In control mice, EE increased neurogenesis but improved only slightly spatial memory performance, a result that agrees with previous studies showing that spatial memory is associated with enhanced levels of hippocampal neurogenesis in aged rats but not in young rats [22].

This study and others using BrdU and Dcx labelling strongly demonstrate reduced number of adult newborn neurons in APP mice [27], [29]–[32]. By contrast, other studies have shown increased neurogenesis in APP_Sw,Ind_ mice 3–7 days after BrdU labeling [25], [28], but reduced survival of newborn neurons can compensate for their over-production detected one week after BrdU administration [25], [28]. Accelerated neuronal differentiation, which induces a temporal increase in the population of newly-generated neurons but reduces their dendritic length and also affects spatial memory [12], may explain the discrepancy of the different studies. To confirm the data obtained with BrdU/NeuN staining we also quantified the number of cells expressing Dcx, a marker of new generated neurons expressed till one month after birth [46]. Our analysis demonstrated reduced number of Dcx proliferating (AB type) and most mature (EF type) Dcx neurons in APP_Sw,Ind_ mice. Short-term EE increased the number of EF type Dcx neurons in APP_Sw,Ind_ mice. Interestingly, reducing the population of newborn neurons at an immature stage, similar to that of EF Dcx neurons, has been demonstrated to impair the formation of long-term spatial memory [15]. It is tentative to speculate that recovery of spatial memory in APP_Sw,Ind_ mice by EE could be related to an increase in the number of most mature Dcx neurons. It should noticed that the number of NeuN/BrdU cells, which are usually considered mature neurons [5]–[7], [15], [46], are similar in EE APP_Sw,Ind_ and control mice, suggesting that an increase of mature newborn neurons must be involved in the recovery of spatial learning and memory in APP_Sw,Ind_ mice. Besides neurogenesis, novelty and enriched environments cause functional and plasticity changes in the brain including among others network reorganization, synaptogenesis, dendritic arborization, angiogenesis, gliogenesis and neurotrophic factors release [16], [17], which may have also contributed to ameliorate cognitive dysfunction in APP mice. For instance, enrichment has been reported to increase expression of genes associated with learning and memory, neurogenesis and cell survival as well as to enhance axonal and long-term potentiation in APP/PS1Δ9 mice [50], [76]. Similarly, long-term EE improves memory and reduces astrogliosis and brain degeneration in a mouse model lacking the presenilin genes [77].

Previous studies have shown dendritic alterations in the brain of AD transgenic mice and patients [36], [53], [54], [78], [79]. Similar to our observations in APP_Sw,Ind_ mice, Wu et al. described a change in the dendritic morphology of DG neurons in PDAPP transgenic mice prior to Aβ deposition [54]. Although the mechanisms underlying changes in the dendritic morphology of DG granule cells before plaque formation remain to be elucidated, elevated levels of soluble oligomeric Aβ species could account for dendritic atrophy [80], [81]. Reduced dendrite growth in AD has been also related to loss of entorhinal projections to the molecular layer of DG and lack of input signal [82], [83]. Importantly, we show that short-term EE in APP mice recovers normal dendritic morphology of Dcx cells, an effect previously observed in a mouse model of Huntington's disease [51]. Notably, total soluble Aβ levels were not changed by short-term EE, indicating that EE could exert beneficial effects on memory and adult neurogenesis in APP_Sw,Ind_ mice independently of Aβ. These data contrast with previous results showing changes of Aβ levels and/or plaques in the hippocampus of APP mice after EE [33], [84].

In summary, this study demonstrates that short-term EE improves hippocampal-dependent learning and memory, and recovers different features of adult neurogenesis including number, maturation and CA3 projections of newborn neurons in APP_Sw,Ind_ mice. The therapeutic benefits of EE on hippocampal-dependent spatial memory are associated with increased of adult neurogenesis and incorporation of new generated neurons into spatial memory circuits in this AD mouse model. Indeed several factors that increase adult neurogenesis such as nerve growth factor, glatiramer acetate, cerebrolyisin or physical exercise have been reported to improve memory in AD patients or animal models [58]. Therefore, we propose that the benefits of acute cognitive stimulation on adult neurogenesis and memory should be considered in future therapeutic strategies in AD.

## Supporting Information

Figure S1 **Animal housing conditions.** Picture showing the environmental enriched (left) or standard (right) housing cages. Numbers above the picture indicate the floor area of each cage.
(TIF)

Figure S2 **Correlation of adult neurogenesis and memory in APP mice.** Correlation plot analysis of memory index taken as percentage of time in the target quadrant during the probe trial in the MWM vs. number of BrdU/NeuN cells in WT and APP_Sw,Ind_ mice. Memory index correlated with the number of new generated neurons in APP_Sw,Ind_ mice (r^2^ = 0.3067; *p* = 0.0085) but not in WT mice (r^2^ = 0.05728; *p* = 0.2895).
(TIF)
