# Supplementary figures and images for: Short-Term Environmental Enrichment Rescues Adult Neurogenesis and Memory Deficits in APPSw,Ind Transgenic Mice
Source: PLoS One. 2011 Feb 9;6(2):e16832. doi: 10.1371/journal.pone.0016832 (PMC3036721; doi:10.1371/journal.pone.0016832)

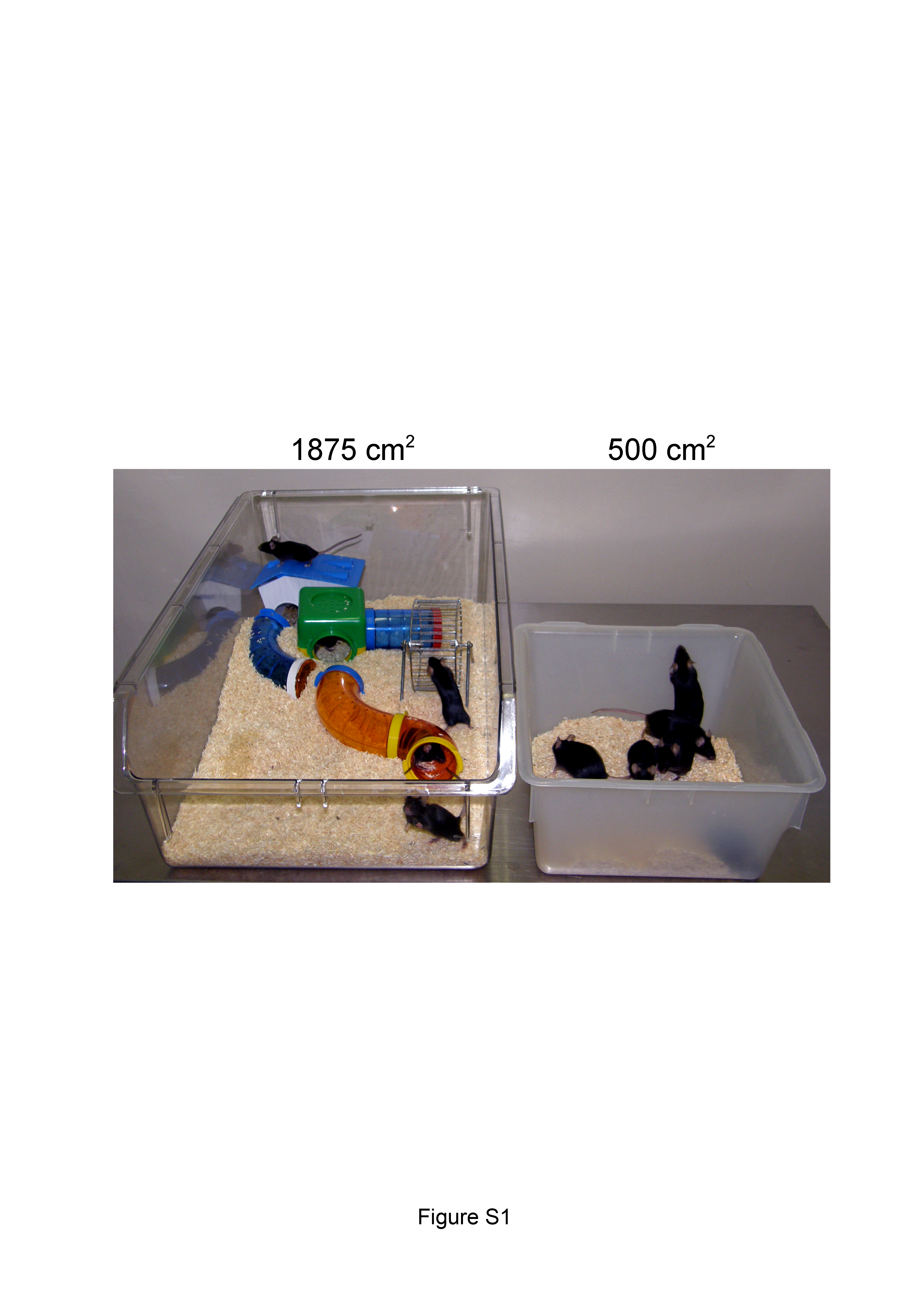

Supplement: Figure S1 — Animal housing conditions. Picture showing the environmental enriched (left) or standard (right) housing cages. Numbers above the picture indicate the floor area of each cage. (TIF) [file pone.0016832.s001.tif]

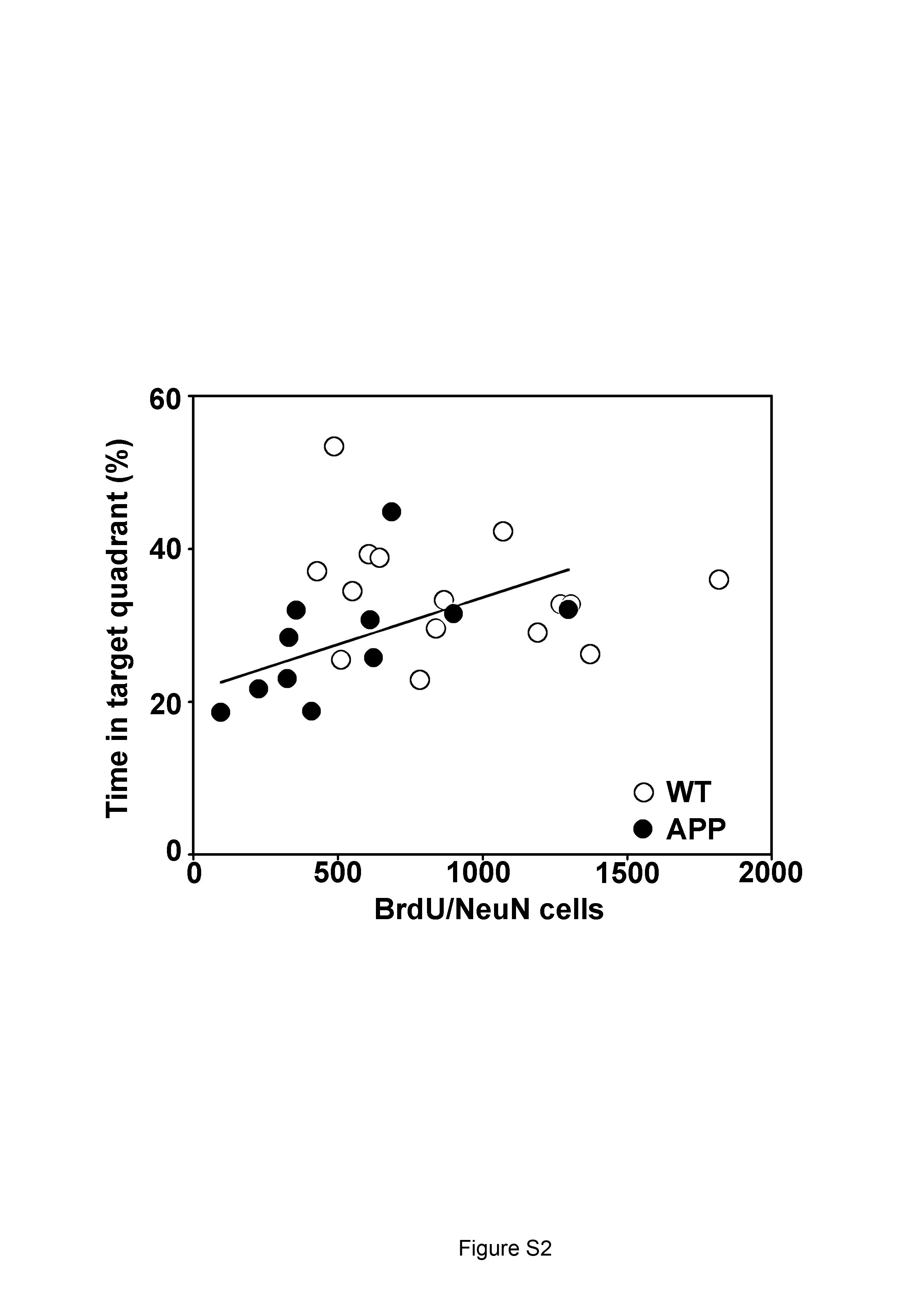

Supplement: Figure S2 — Correlation of adult neurogenesis and memory in APP mice. Correlation plot analysis of memory index taken as percentage of time in the target quadrant during the probe trial in the MWM vs. number of BrdU/NeuN cells in WT and APPSw,Ind mice. Memory index correlated with the number of new generated neurons in APPSw,Ind mice (r2 = 0.3067; p = 0.0085) but not in WT mice (r2 = 0.05728; p = 0.2895). (TIF) [file pone.0016832.s002.tif]
